# The road to deforestation: Edge effects in an endemic ecosystem in Sumatra, Indonesia

**DOI:** 10.1371/journal.pone.0217540

**Published:** 2019-07-01

**Authors:** Erin E. Poor, Virta I. M. Jati, Muhammad Ali Imron, Marcella J. Kelly

**Affiliations:** 1 Department of Fish and Wildlife Conservation, Virginia Tech, Blacksburg, Virginia, United States of America; 2 World Wildlife Fund, Pekanbaru, Riau, Indonesia; 3 Department of Forest Resources Conservation, Univesitas Gadjah Mada, Yogyakarta, Indonesia; Michigan State University, UNITED STATES

## Abstract

Worldwide, roads are a main driver of deforestation and degradation as they increase forest access along the forest edge. In many tropical areas, unofficial roads go unreported and unrecorded, resulting in inaccurate estimates of intact forested areas. This is the case in central Sumatra, which boasts populations of critically endangered Sumatran elephants (*Elephas maximus sumatrensis*), tigers (*Panthera tigris sumatrae*) and other endemic flora and fauna that make the area globally unique. However, maps do not reflect the reality of forest loss in the area. Here we present new maps from 2002 and 2016 of digitized and ground-truthed roads in one of Sumatra’s unique lowland tropical protected areas, Tesso Nilo National Park. Using our newly created roads dataset, we examine the distribution of forest with respect to distance to roads. Our data show >2,400 km of roads within the national park in 2016 –nearly a 10-fold increase from roads known in 2002. Most forest (82–99%) within Tesso Nilo falls within 100 m, 500 m, and 1000 m of road edges. Length of road increased 157% and road density increased from 1.06 km/km^2^ to 2.63 km/km^2^ from 2002–2016. Our results suggest that this endemic ecosystem is facing substantial threat from roads and their associated impacts. Without swift management action, such as road closures and increased enforcements by park management, this ecosystem, and its endemic wildlife, could be lost. It is imperative that protected areas worldwide more rigorously consider roads and road effects on ecosystem fragmentation in their conservation plans.

## Introduction

Globally, as forests are fragmented into smaller patches, biodiversity is lost directly as a result of forest clearing, and indirectly through increased forest access and illegal use of wildlife resources. In the Amazon, extinction rates are negatively correlated with area of forest fragments [1, 2] and fragments 0.01–0.1 km^2^ in size lose species across taxa at a higher rate than fragments 1 km^2^ or greater [3–6]. Furthermore, biodiversity is not only affected by the size of remnant forest patches, but also by the distance to, and habitat between neighboring forest patches, which can play roles in biodiversity persistence in a disturbed landscape [7, 1]. Thus, maintaining intact forested areas that are connected to other forested areas across a landscape is integral to maintaining global tropical biodiversity.

Roads and trails are factors that contribute to fragmentation of forests, and there is ample evidence that roads have an overall negative affect on wildlife populations across taxa and ecosystems due to increased traffic, noise and light pollution, and human access [8]. Such effects are often measured by wildlife population abundance, species richness, or home range activity in relation to road density or distance from a road. For example, grizzly bears (*Ursus arctos horribilis*), cougars (*Puma concolor*) and wolves (*Canis lupus*) have been found to select home ranges with significantly lower road density compared to road density in the surrounding landscapes [9–11]. Other species such as plains zebras (*Equus quagga*), elephants (*Loxodonta africana*), wildebeests (*Connochaetes* sp.), elands (*Taurotragus oryx***)**, jackals (*Canis* sp.) [12], salamanders, amphibians [13, 14], tropical understory birds [15] and rodents [16], and tropical forest elephants (*L*. *cyclotis*) [17] showed reduced animal sign, movements, and/or reduced abundance within a certain distance from roads, indicating that roads negatively affect habitat use by, or demography of, these taxa. In the tropics in particular, wildlife that has adapted to specialized niches may be negatively impacted by increased hunting brought by roads and trails [18], pollution and runoff from roads [19], fragmentation of populations within forest patches [20, 21], structural changes in forest edges along roads [22] that lead to changes in microclimate [23], and a variety of other impacts (see Laurance et al. [24] for a more complete review). In Southeast Asia, a variety of experts agree that roads impact wildlife by increasing access to forests and increasing deforestation of wildlife habitat [25].

While protected areas may, in some cases, slow deforestation and confer protection within their borders [26], protected areas are not immune to the effects of deforestation that occur outside their borders. Decreasing forest, increasing logging, and increasing fires outside of protected areas have been shown to negatively affect tropical forest protected area health (as measured by expert opinion on change in guild abundance) in 60 randomly selected protected areas [1]. Thus, as forest surrounding a protected area decreases, wildlife populations within the protected area may decline as well. If little natural forest remains outside of a protected area and the distance between neighboring patches is greater than a species’ dispersal ability, these species may have low long-term persistence [27], ultimately resulting in decreased wildlife populations inside and outside of protected areas. While factors outside of protected areas were the most important factors negatively affecting protected area health, decreasing forest area within a protected area, increasing hunting, and increasing logging were also important drivers of protected area health [1].

In Indonesia, a tropical archipelago spanning the Sundaland and Wallacea biodiversity hotspots [28], many protected areas have been degraded due to the expansion of oil palm plantations in the past 20 years [29]. Indonesia contains 10% of the world’s plant species and 17% of the world’s bird species [30], and recently eclipsed Brazil as having the highest deforestation rate in the world [31]. Indonesia’s protected area system was established at the beginning of the 20^th^ century to preserve some of the world’s highest diversity forests and unique species such as critically endangered Sumatran elephants (*Elephas maximum sumatrensis*) and Sumatran tigers (*Panthera tigris sumatrae*), which can be found in Tesso Nilo National Park in Riau, Sumatra. Border protection in many Indonesian protected areas is not well enforced and protected areas are easily accessed by the local human population that hunts wildlife, tends oil palm plantations, and harvests timber and non-timber forest resources via roads and narrower foot and motorbike trails [32–34]. While Sunarto et al. [35] documented a decline in human activity in Tesso Nilo from 2005–2011 possibly due to PA establishment in 2004, remote cameras still had a capture rate of seven photos of humans per 100 trap nights.

As a former logging concession, Tesso Nilo can be accessed through small roads or pathways on motorbike or foot, most of which are not present in Tesso Nilo’s official roads documentation. While the impacts of these smaller roads may be less than those from highways that include wide clearings, the impacts of access trails (where canopy may be intact) and larger roads in this landscape have yet to be studied, and the increased access to the forest from such roads could be contributing to biodiversity loss within this unique and endemic eco-floristic zone [36]. In this study, we aim to 1) determine if, and how much roads within the park have increased from 2002–2016 with respect to length and density, and 2) determine the amount and distribution of Tesso Nilo’s remaining natural forest with respect to roads.

## Methods

### Study area

**Fig 1:**
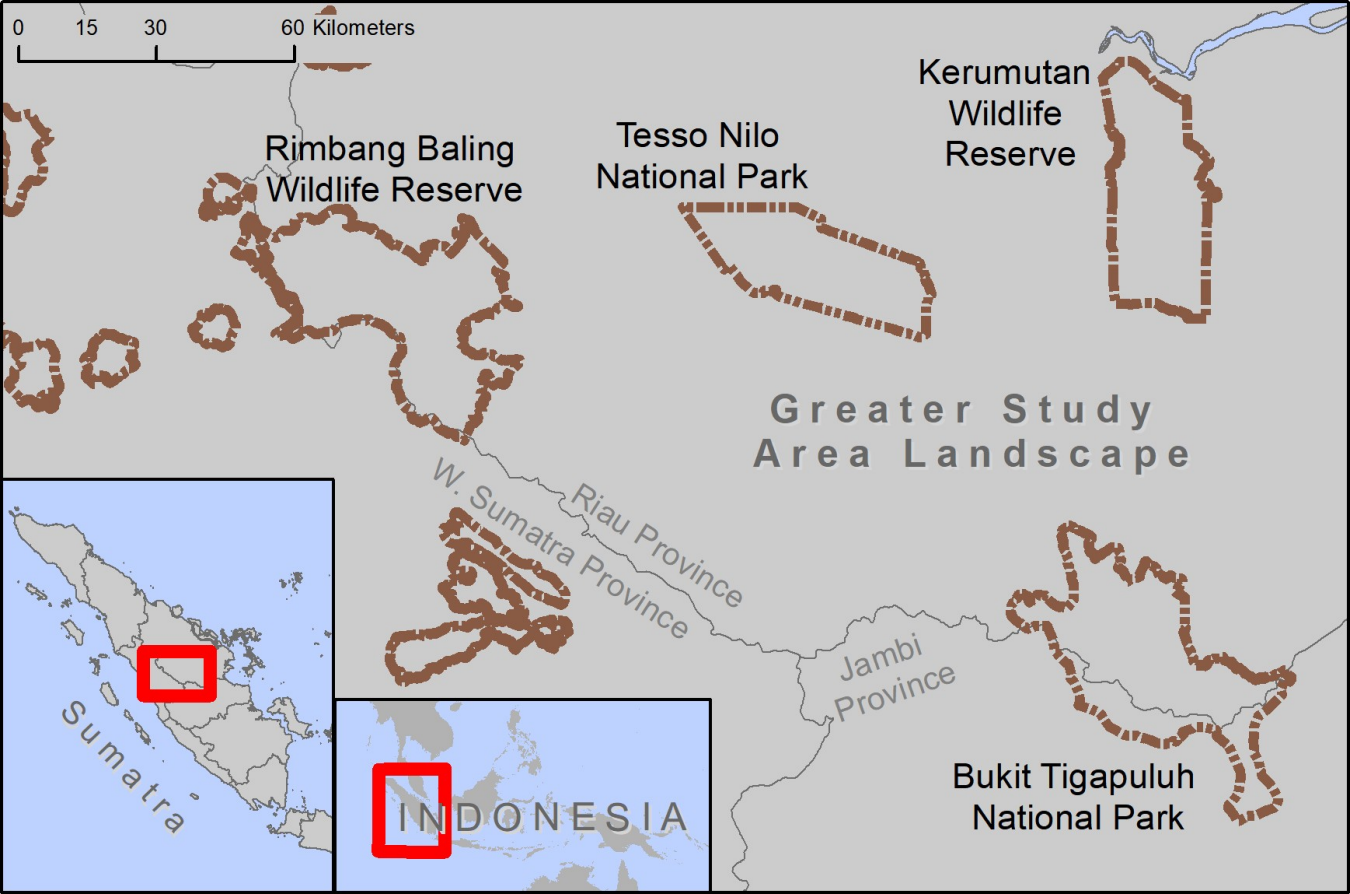
Study area. Location of the greater study area landscape (top), and Tesso Nilo National Park in relation to nearby protected areas within central Sumatra, Indonesia.

Tesso Nilo National Park was established in 2004 in Riau Province (pop. 6.3 million) (Fig 1) and contains some of the last remaining lowland tropical rainforest in Sumatra. The climate of Riau is classified in the Koppen-Geiger system as Af, tropical. Average temperature is 27° C while average rainfall is 2696 mm per year. The park, initially 386 km^2^, was expanded to 830 km^2^ in 2009 to better protect populations of Sumatran elephants and the endemic floristic community found within the park. Formerly a group of multiple adjacent timber concessions, Tesso Nilo was established with the intent to end the rapid deforestation that was beginning to occur, and to curb poaching of tigers and elephants as well as reduce human-wildlife conflict by providing a refuge for wildlife. Although community managers were involved in its foundation, indicating support for the protected area, there has been a lack of strict protection and insufficient patrol measures against encroachment of the park, which has likely become worse with the increased prevalence of oil palm in the region [37]. Tesso Nilo National Park is categorized Class III (of five) Tiger Conservation Landscapes (TCL) meaning it has habitat to support some tigers but also has moderate to high levels of threat and minimal conservation investment [38]. Tesso Nilo National Park is situated within in a broader landscape (Fig 1) that includes potential tiger habitat in Bukit Tigapuluh National Park to the south, Rimbang Baling Wildlife Reserve to the west, and Kerumutan Wildlife Reserve to the east. There could be a possibility of maintaining viable tiger populations in Tesso Nilo National Park if it remains connected to Bukit Tigapuluh National Park and Rimbang Baling Wildlife Reserve, as both have mountainous connections to the inaccessible Bukit Barisan Mountains on Sumatra’s western edge. This landscape is considered vitally important in retaining west-east dispersal connection for tigers across Sumatra.

### Road length and density

We digitized all roads identifiable within Tesso Nilo in 2002 and 2016 using composite infrared Landsat images and Digital Globe World View 2. We used these two years due to availability of land cover data in the study area [37]. To aid in clear identification, all Landsat images were pan-sharpened with band 8 to 15 m accuracy. All geospatial data resulted from digitization projected to 48 S UTM 1984. Land cover data were created by Poor et al. [37]. Additionally, from June–July 2016, we drove roads passable by motorbike within Tesso Nilo using GPS tracking to further identify roads and aid in creating the new roads data. We drove most roads within the forested area of Tesso Nilo, but were unable to drive all roads within the park due to contested areas in illegal palm oil plantations. We attempted to digitize roads regardless of size, from approximately 0.25 m in width or passable only by foot, to larger 3-meter-wide roads that are passable by car on terrain such as packed gravel to loose dirt and vegetation. We do not distinguish between large and small roads.

To determine the density of roads, line density (kilometers of roads per square kilometer) was calculated using a 1 km radius at 30 m resolution. Analysis was restricted to within park boundaries so incomplete roads data outside of Tesso Nilo would not affect road density calculations.

### Potential impacts on forest distribution

Based on our literature review, we determined that specific taxa would be affected by road noise, light, temperature change or other effects at different widths and thus we chose 100 m (small carnivores and rodents, [39]; birds, [14], amphibians [14, 40]), 500 m (mid-sized carnivores, [41] and 1000 m (ungulates [39, 42]) from roads to account for impacts of forest loss and edge effects due to roads on various species. While we recognized that tropical ecosystems and species may vary in their response to traffic on roads, research on specific distances affecting tropical species is relatively rare. Thus, we use these road impact distances more as a general guide for how much forest may be affected by roads. We created buffers around the newly created GIS roads dataset and subsequently removed these buffer areas from the forested area of Tesso Nilo using GIS, to identify ‘core’ forest areas [43].

To identify natural forest patches we used 2002 and 2016 land cover data newly created for this area [37] and extracted land cover classes of ‘natural forest’ (primary or secondary), based on the methods used by Poor et al. [37]. Plantations are not included in this category. The area of each forest patch was then calculated and the distance from each patch edge to the closest neighboring forest patch edge was also calculated using Euclidean distance. We did this for forest patches across the greater Riau Province landscape (Fig 1), including Tesso Nilo, as well as for forest within Tesso Nilo, to determine how the forest distribution within Tesso Nilo compares to the landscape as a whole. Roads were not incorporated into the forest patch calculations of forest outside of Tesso Nilo due to inconsistent roads data across the landscape. All spatial data were projected to UTM Zone 48N and analyses were completed using ArcGIS 10.3 [44] and pixel size and minimum mapping unit were 30 m.

## Results

### Road length and density

**Fig 2:**
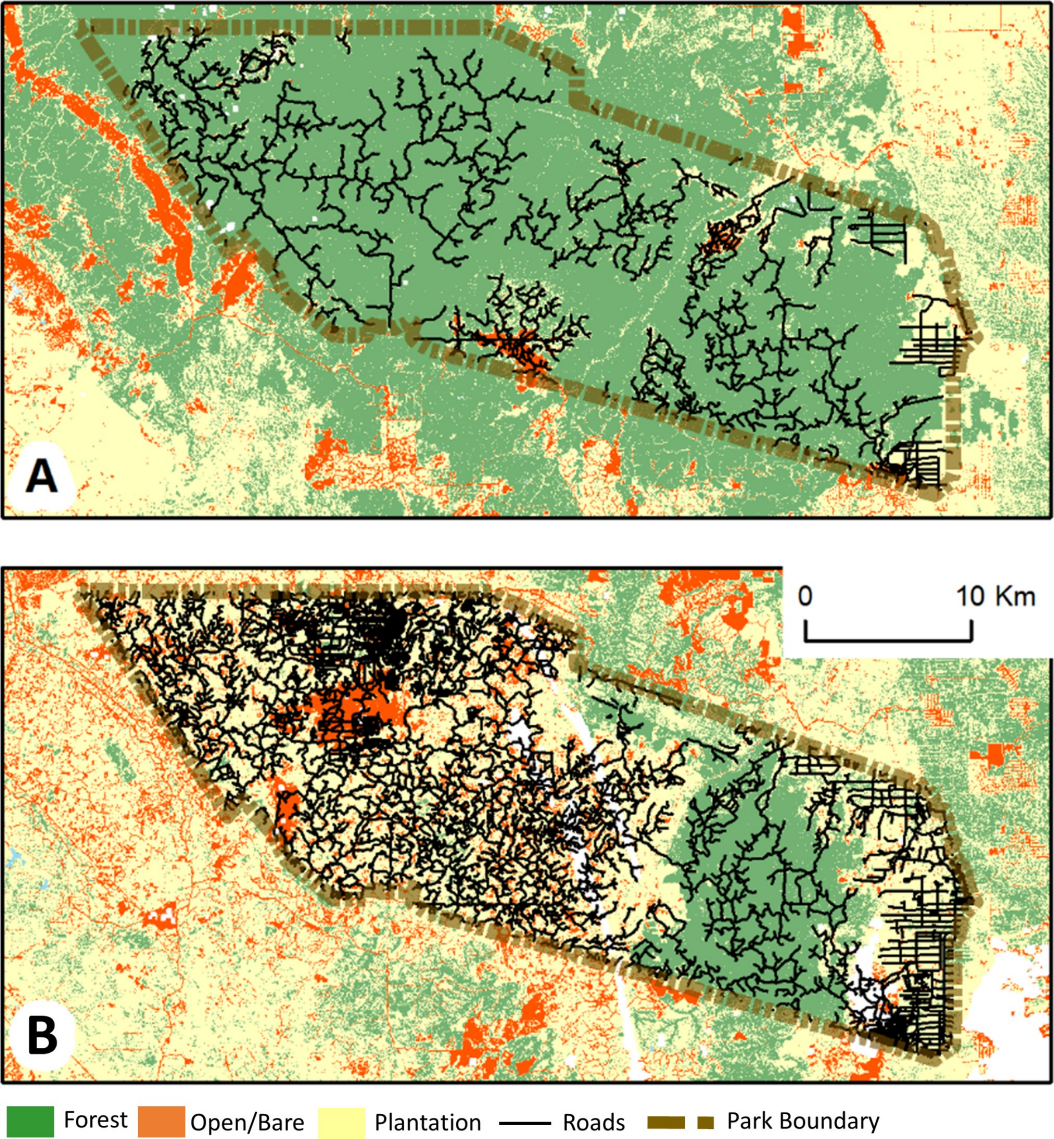
Mapped roads in Tesso Nilo National Park. Roads (without impact buffers) and land cover (Yellow–plantation, Orange–bare land, Green–forest) within Tesso Nilo National Park, Riau, Sumatra in 2002 (A), and 2016 (B). Land cover from Poor et al. 2019.

In 2002, we identified 954 km of previously unrecorded roads via digitization within Tesso Nilo, 321.32 km of which were within forested areas (Fig 2). In 2016, we found 2,484 km of roads within Tesso Nilo (Fig 2). In 2002, average road density within the entire park was 1.06 km road/km^2^, while density increased to 2.63 km road/km^2^ in 2016. Within just the natural forest of Tesso Nilo, average road density more than doubled from 0.41 km road/km^2^ to 0.88 km road/km^2^ during the 14 year period of the study.

### Potential impacts on forest distribution

**Fig 3:**
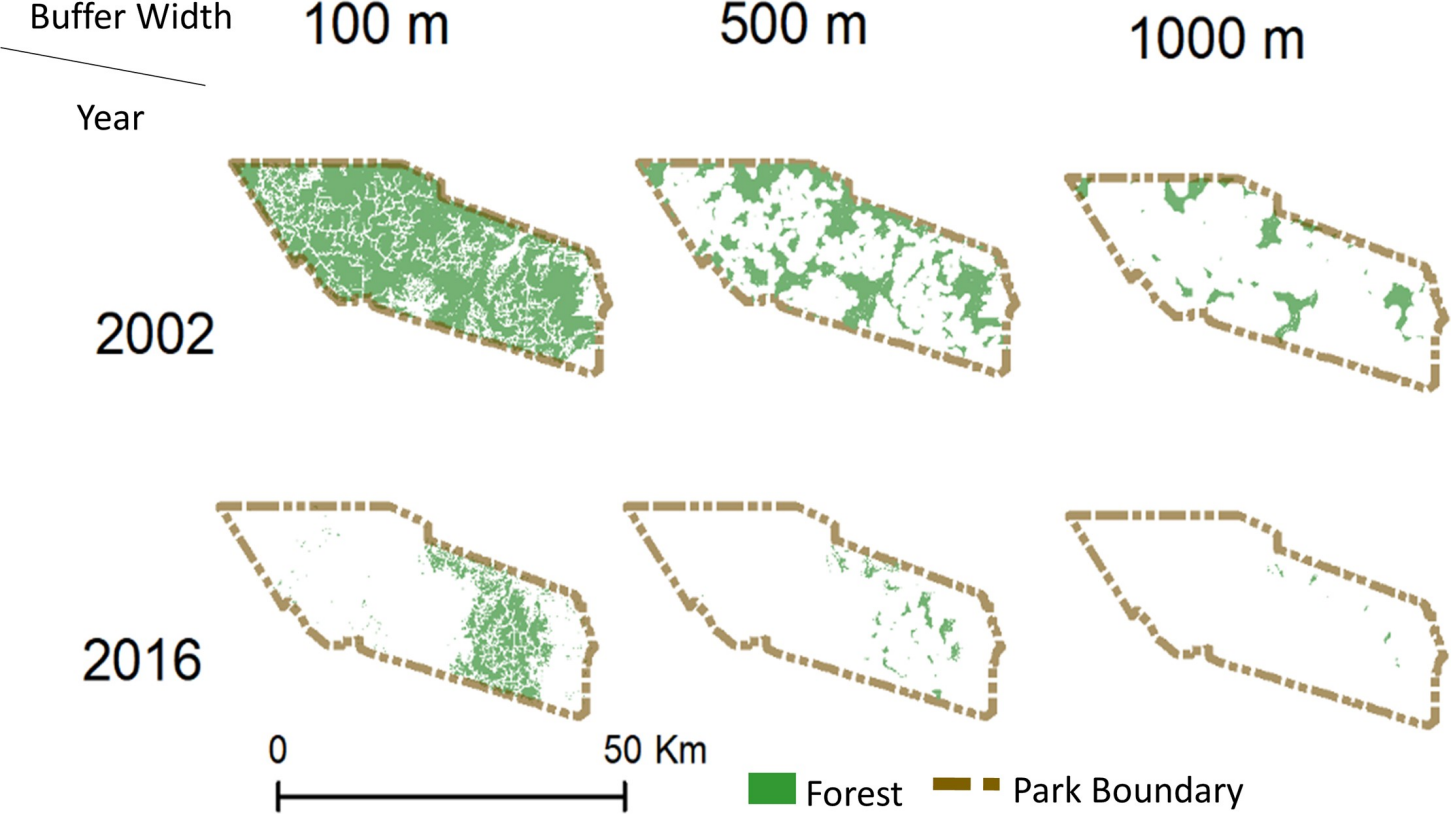
Forest and road impact buffers. Natural forest distribution in Tesso Nilo National Park for 2002 and 2016 within varying distances from roads: 100 m, 500 m, and 1000 m. Land cover from Poor et al. 2019.

**Fig 4:**
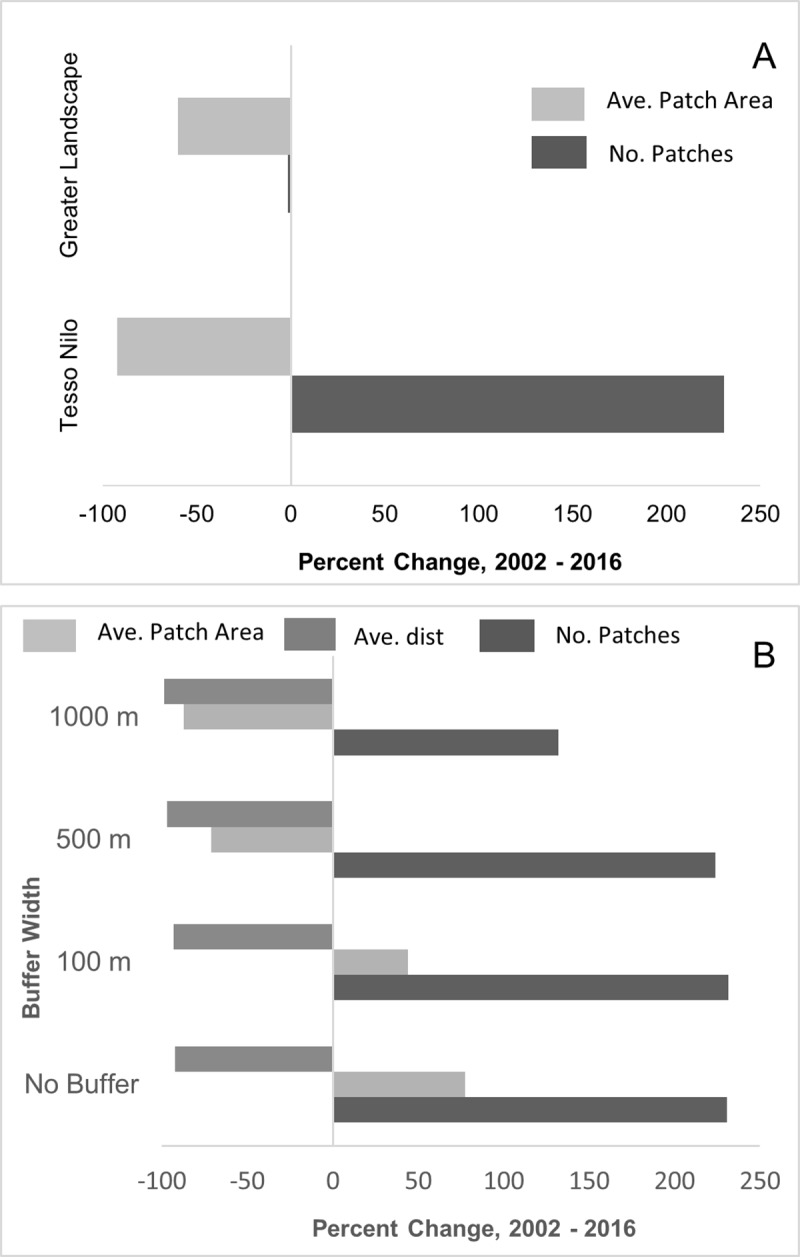
Changes in forest distribution. Percent change in average forest patch area (light gray) and the number of forest patches (dark gray) in Tesso Nilo and the greater landscape (A), and percent change in number of forest patches (dark gray), average forest patch area (light gray) and average distance to neighboring forest patches (medium gray) at different road impact widths (B) within Tesso Nilo.

Without incorporating roads into forest area calculations, natural forest in Tesso Nilo decreased 76% from 689 km^2^ to 163 km^2^ during the study period (Table 1). When a 100 m buffer is used to model edge effects, the amount of natural forest decreased by 80% during the study period (Table 1; Fig 3). The 1000 m road effects buffer reduced forest in Tesso Nilo 97% from 72 km^2^ in 2002 to 2 km^2^ in 2016, indicating that if road impacts permeate 1 km into Tesso Nilo’s forest, only 3% of forest will be unaffected. All modeled edge effect buffers decreased total forest area and forest patch size, and increased number of forest patches within Tesso Nilo (Fig 3). Distance between patches increased with larger buffer widths (Table 1). Throughout the broader landscape (Fig 1), including Tesso Nilo, there were 409,602 forest patches with a mean size of 0.05 km^2^ compared to 403,414 natural forest patches with an average size of 0.02 km^2^ in 2016 (Fig 4) Total natural forest decreased from 20,837 km^2^–10,198 km^2^. Average distance from a forest patch within Tesso Nilo to the nearest patch outside Tesso Nilo remained nearly 2 km.

**Table 1:** Changes in forest with edge effect buffers. Amount of forest, number of patches, distance between forest patches (± SD), patch size (± SD), and taxa expected to be impacted by edge effects within Tesso Nilo National Park (TNNP) when taking roads into account at different buffer widths.

			Edge Effect					
	0 m		100 m		500 m		1000 m	
	2002	2016	2002	2016	2002	2016	2002	2016
Total mount of TNNP Forest (km^2^)	688.3	162.9	589.8	118.5	233.3	22.3	72.2	2.1
Number of Patches	1305	4313	1002	3321	224	726	47	109
Average Distance Between Patches (m)	21 (±51)	38 (±95)	35 (±69)	50 (±117)	2574 (±4050)	74 (±479)	1041 (±1227)	134 (±732)
Average Patch Size (m^2^)	520,000 (±18,000,000)	40,000 (±2,160,000)	590,000 (±11,600,000)	40,000 (±19,000,000)	1,040,000 (±6,000,000)	30,000 (±240,000)	1,540,000 (±3,640,000)	20,000 (±70,000)
Taxa affected			Small carnivores, birds, amphibians		mid-sized carnivores		Ungulates	

## Discussion

The decrease in forest within Tesso Nilo National Park due to roads and access trails increases the importance of natural forest areas outside of the protected area to provide habitat, corridors, and stepping stones for wildlife that face habitat loss within the park. It is widely recognized that Tesso Nilo has suffered from deforestation and degradation [37, 45, 46], but this is the first study in Indonesia that has quantified the effects of increases in road density on forest within a protected area. This research was motivated by receiving a roads dataset from the government of Indonesia that did not appear to reflect the on-the-ground situation, and we determined it necessary to create an updated and verified roads dataset. Furthermore, no previous research has focused on the impacts of human activity or roads in Tesso Nilo despite the declining populations of tigers [35] and elephants and the anecdotal evidence of recent increased human activity in the park. Given the evidence that apex predators and large vertebrates are the most disturbance-sensitive species in protected areas in the tropics [1], it is important to understand the impact roads have on multiple critically endangered species within Tesso Nilo, and to identify areas where impacts could be mitigated. We were unable to assess direct impacts to biodiversity during this study, but other studies have shown the negative impacts of road effects and human activity on tropical flora and fauna [8, 24, 29].

From our digitization, it is clear that roads within Tesso Nilo have increased greatly, likely due to increased oil palm expansion within the park [37]. In 2016, nearly all of the natural forest within the protected area was within a km of a road, and thus within human reach. Prior research in temperate regions shows that road densities greater than 0.38 km/km^2^ negatively affected carnivore abundance and behavior [47, 48], but the impacts of road density vary by species and by road or trail type. Research on the impacts of road density in tropical systems is lacking, but given that most of Tesso Nilo natural forest is within 1 km of a road, and the road density of 2.63 km road/km^2^ far exceeds other densities that have been shown to affect biodiversity, roads are likely to have negative impacts on most wildlife species in Tesso Nilo.

We note that here we address ‘roads’ as the passable width along a path or trail, and do not consider canopy or forest width clearing. Effects of roads or trails can be impacted by differences in cleared width as well. However, trails–those paths that are too narrow to allow commercial vehicle access to forest interiors and may still have intact canopy–can still allow forest access via foot, bicycle, or motorbike but may have less of an impact on forest structure. In northern Sumatra, the number, diversity and evenness of species were lower in a high human foot traffic area versus a low traffic area and wildlife altered activity patterns to avoid human activity, even though the roads were not accessible by vehicle [49]. However, we recommend examining effects on canopy dwelling species and forest structure as part of any future research.

From 2002–2016, without taking road buffer effects into account, 76% of natural forest area within Tesso Nilo was lost. The remaining natural forest area is not large enough to support more than one tiger, notwithstanding use of non-forested areas. In addition, average patch size decreased and forest patches within the park are likely losing species at an increased rate compared to intact forest due to small forest patch size [3–6]. From 2002 to 2016, patch size within Tesso Nilo decreased 92% and average patch size of forest outside of Tesso Nilo decreased by 60%. When conservative road effects (100 m buffer) are taken into account, the 2002 average patch size (i.e. core forest areas) increased 13% and the 2016 average patch size decreased slightly. The increase in size seen in 2002 is due to smaller patches overlapping areas within the 100 m road buffer, thus being removed from the calculations. Though the 2016 total amount of forest may be large enough to support wildlife, the small average patch size in 2016 indicates a highly fragmented landscape. When less conservative road effects are taken into account (500 m or 1000 m buffers), there is very little forest remaining, and that which remains is likely not suitable for wildlife in the long-term.

Forest outside of the protected area also decreased but not as much as forest within the park in 2002, possibly due to the broader landscape’s already degraded state. While small, the forest loss outside of Tesso Nilo is alarming because, if little natural forest remains outside of Riau’s protected areas, and the distance between neighboring forest patches is greater than a species’ dispersal ability, these species may have low long-term persistence [27]. Forest patches in Tesso Nilo are now >2 km from the closest forest patch outside of the park, which may be farther than some insect, small mammal and amphibian dispersal distances. This distance limits patch usefulness as stepping stones between protected areas. On the other hand, forested areas are within 0.01 km from, or nearly adjacent to, oil palm plantations. While some species may be able to persist by using plantations as habitat or stepping stones [50], we may be at risk of losing lowland Riau’s unique assemblage of biodiversity.

To improve our newly created roads layers, we attempted to ground-truth the data by driving many of the roads within Tesso Nilo. However, some areas of Tesso Nilo have been illegally converted to oil palm and ownership is highly contested, thus the field team was unable to survey these areas. Nevertheless we were able to combine ground-truthing with GIS layers to substantially increase the veracity of the road layers map. We digitized roads using Google Earth imagery, but visibility in some images was restricted by smoke from slash and burn agriculture or cloud cover. Despite our limitations, our new digitized road maps provide fresh insight into the high level of disturbance occurring in and around Tesso Nilo National Park. Since these roads are not officially documented by the government, and there is very little on-the-ground enforcement, Tesso Nilo may lose even more forest through the access these roads provide and due to the lack of protected area law enforcement.

It is clear that, though often used as an example of protected lowland forest, due to uncontrolled development of roads, the forest in Tesso Nilo is now highly fragmented and likely impaired due to increased edge effects and impacts of degradation from outside of the park. The park is also fairly isolated from nearby unprotected forest. Tesso Nilo is not providing the protection it was intended to, and is likely acting only as a ‘paper park’. The unique lowland Eastern Peneplain eco-floristic sector, one of 38 unique zones in Sumatra identified by floral and geologic features, previously covered a large portion of Riau is now listed as critically endangered and only remains within Tesso Nilo National Park [36]. The most recent estimates (2007) indicate >70% of this zone has been lost [36], and today, given the further deforestation and degradation we have documented within Tesso Nilo, it is likely that very little of this zone remains. Unfortunately, few published studies have focused on cataloging or quantifying changes in the insect, bird, small mammal, amphibian, and reptile communities that inhabit this lowland area of Sumatra, and we suggest cataloging and researching this area as soon as possible.

Continued loss of Tesso Nilo, its endemic biodiversity, and its wildlife of global importance such as Sumatran elephant and tiger, could result in reduced wildlife dispersal across greater Riau, and an increased chance of isolation and extinction in the near future [48], but efforts to conserve Tesso Nilo should continue because there is evidence that wildlife can use the small patches of forest in and around Tesso Nilo [51] even if the forests of the park cannot be fully restored. In addition, the government of Indonesia recently launched a plan to restore Tesso Nilo by removing illegal oil palm plantations and engaging locals in more sustainable farming. Protected areas are often more effective when local communities are included in decision making, and increasing community engagement is likely key to the success of Tesso Nilo. Future research should include comparisons of similar landscapes that have successfully enforced protections.

If deforestation and degradation within Tesso Nilo continue, it is likely this entire eco-floristic zone, any benefits it provides as a refuge for climate change or from poaching, and the endangered endemic wildlife species that inhabit it, will be lost [45]. When forest assessments fail to take road effects into account and road-building does not adhere to ecologically sustainable principles [52], Tesso Nilo and other tropical protected areas may appear to be ecologically healthier and larger than they actually are. Roads can have potentially far-reaching and substantial negative impacts on forest extent and distribution that are often not taken into consideration–especially when roads are small, or in systems that are thought to be ‘intact’ [53]. Quantifying the effects of roads on forest distribution and working to decrease human activity and mitigate road impacts in this unique, and other understudied systems similar to that of Tesso Nilo National Park and the surrounding system, should be a global conservation priority.

## References

[1] Laurance WF, Useche DC, Rendeiro J, Kalka M, Bradshaw CJA, Sloan SP, et al Averting biodiversity collapse in tropical forest protected areas. Nat. 2012, 489: 290–294. 10.1038/nature11318 22832582

[2] MacArthur RH, Wilson EO. The theory of island biogeography. Princeton, New Jersey: Princeton University Press; 1967.

[3] Laurance WF, Albernaz AKM, Schroth G, Fearnside PM, Bergen S, Venticinque EM, et al Predictors of deforestation in the Brazilian Amazon. J Biogeogr. 2002; 29: 737–748.

[4] Lovejoy TE, Bierregaard RO Jr, Rylands AB, Malcom JR, Quintela CE, Harper LH, et al Edge and other effects of isolation on Amazon forest fragments In Soule M.E. editor. Conserv Biol: The science of scarcity and diversity. Sunderland, Massachusetts: Sinauer Associates; 1986 pp 257–285.

[5] Harper LH. The persistence of ant-following birds in small Amazonian forest fragments. Acta Amazon.1989; 19: 249–263,

[6] Brown KS Jr, Hutchings RW. Disturbance, fragmentation, and the dynamics of diversity in Amazonian forest butterflies In Laurance WF, Bierregaard Jr RO, editors. Tropical forest remnants: ecology, management, and conservation of fragmented communities. Chicago: University of Chicago Press; 1997 pp 91–110.

[7] Gascon C, Lovejoy TE, Bierregaard RO Jr, Malcolm JR, Stouffer PC, Vasconcelos HL, et al Matrix habitat and species richness in tropical forest remnants. Biol Conserv. 1999; 91(2–3): 223–229.

[8] Fahrig L, Rytwinski T. Effects of roads on animal abundance: An empirical review and synthesis. Ecol Soc. 2009; http://www.ecologyandsociety.org/vol14/iss1/art21/Studies.

[9] Mace RD, Waller JS, Manley TL, Lyon LJ, Zurring H. Relationships among grizzly bears, roads, and habitat in the Swan Mountains, Montana. J Appl Ecol. 1996; 33: 1395–1404.

[10] Van Dyke FG, Brocke RH, Shaw HG. Use of road track counts as indices of mountain lion presence. J Wildl Manage. 1986; 50: 102–109.

[11] Mech LD, Fritts SH, Radde GL, Paul WJ. Wolf distribution and road density in Minnesota. Wildl Soc Bull. 1988; 16: 85–87.

[12] Newmark WD, Boshe JI, Sariko HI, Makumbule GK. Effects of a highway on large mammals in Mikumi National Park, Tanzania. Afr J Ecol. 1996; 34:15–31.

[13] Porej D, Micacchion M, Hetherington TE. Core terrestrial habitat for conservation of local populations of salamanders and wood frogs in agricultural landscapes. Biol Conserv. 2004; 120: 399–409.

[14] Hoskin C, Goosem M. Road impacts on abundance, call traits and body size of rainforest frogs in north-east Australia. Ecol Soc. 2010; 15 (3): 15.

[15] Laurance S. Responses of understory rain forest birds to road edges in central Amazonia. Ecol Appl. 2004; 14: 1344–1357.

[16] Goosem M. Effects of tropical rainforest roads on small mammals: fragmentation, edgeeffects and traffic disturbance. Wildl Res. 2002; 29(3): 277–289.

[17] Blake S, Deem SL, Strindberg S, Maisels F, Momont L, Isia I-B, et al Roadless wilderness area determines forest elephant movements in the Congo basin. PLOS ONE. 2008; 3: e3546 10.1371/journal.pone.0003546 18958284 PMC2570334

[18] Laurance WF. Catastrophic declines of Australian rainforest frogs: Is unusual weather responsible? Biol Cons. 1996; 77(2–3): 203–212.

[19] Pratt C, Lottermoser BG. Mobilisation of traffic-derived trace metals from road corridors into coastal stream and estuarine sediments, Cairns, northern Australia. Environ Geol. 2007; 52: 437–448.

[20] Goosem M. Fragmentation impacts caused by roads through rainforests. Curr Sci. 2007; 93: 1587–1595.

[21] Goosem M. Effects of tropical rainforest roads on small mammals: Edge changes in community composition. Wildl Res. 2000; 27(3): 151–163.

[22] Laurance WF, Lovejoy TE, Vasconcelow HL, Bruna EM, Didham RK, Stouffer PC, et al Ecosystem decay of Amazonian forest fragments: A 22-year investigation. Conserv Biol. 2002; 16: 605–618.

[23] Pohlman C, Turton SM, Goosem M. Edge effects of linear canopy openings on tropical rain forest understory microclimate. Biotropica. 2007; 39(1): 62–71.

[24] Laurance WF, Goosem M, Laurance SGW. Impacts of roads and linear clearings on tropical forests. Trends Ecol Evol. 2009; 24(12): 659–669. 10.1016/j.tree.2009.06.009 19748151

[25] Clements GR, Lynam AJ, Gaveau D, Yap WL, Lhota S, Goosem M, et al Where and how are roads endangering mammals in Southeast Asia’s forests? PLOS ONE. 2014; e115376 10.1371/journal.pone.0115376 25521297 PMC4270763

[26] DeFries R, Hansen A, Newton AC, Hansen MC. Isolation of protected areas in tropical forests over the past twenty years. Ecol Appl. 2005; 15(1): 19–26.

[27] Laurance WF, Ferreira LV, Rankin-de Merona JM, Laurance SG. Rain forest fragmentation and the dynamics of Amazonian tree communities. Ecology. 1998; 79(6): 2032–2040.

[28] Myers N, Mittermeier RA, Mittermeier CG, da Fonseca GAB, Kent J. Biodiversity hotspots for conservation. Nat. 2000; 403: 853–858. 10.1038/35002501 10706275

[29] Kinnaird MF, Sanderson EW, Brien TGO, Wibisono HT, Woolmer G. Deforestation trends in a tropical landscape and implications for endangered large mammals. Conserv Biol. 2010; 17(1): 245–257.

[30] Land Resources Department/Bina program. The land resources of Indonesia: A national overview from regional physical planning program for transmigration. Jakarta, Indonesia: Land Resource Department, Natural Resources Institute, Direktorat Jenderal Penyiapan Pemukiman, Departemen Transmigrasi; 1990.

[31] Margono BA, Potapov PV, Turubanova S, Stolle F, Hansen MC. Primary forest cover loss in Indonesia over 2000–2012. Nat Clim Chang. 2014; 4: 730–735.

[32] Luskin MS, Christina ED, Kelley LC, Potts MD. Modern hunting practices and wild meat trade in the oild palm plantation-dominated landscapes of Sumatra, Indonesia. Hum Ecol. 2014; 42(1): 35–45.

[33] Linkie M, Sloan S, Kasia R, Kiswayadi D, Azmi W. Breaking the vicious circle of illegal logging in Indonesia. Conserv Biol. 2014; 28(4): 1023–1033. 10.1111/cobi.12255 24628366

[34] Harbi J, Erbaugh JT, Sidiq M, Haasler B, Nurrochmat DR. Making a bridge between livelihoods and forest conservation: Lessons from non timber forest products’ utilization in South Sumatera, Indonesia. For Policy Econ. 2018; 94: 1–10.

[35] Sunarto S, Kelly MJ, Parakkasi K, Klenzendorf S, Septayuda E, Kurniawan H. Tigers need cover: multi-scale occupancy study of the big cat in Sumatran forest and plantation landscapes. PloS One. 2012; 7(1): e30859 10.1371/journal.pone.0030859 22292063 PMC3264627

[36] Laumonier Y, Uryu Y, Stüwe M, Budiman A, Setiabudi B, Hadian O. Eco floristic sectors and deforestation threats in Sumatra: Identifying new conservation area network priorities for ecosystem-based land use planning. Biodivers Conserv. 2010; 19(4): 1153–1174.

[37] Poor EE, Shao Y, Kelly MJ. Mapping and predicting forest loss in a Sumatra tiger landscape from 2002–2050. Environ Manage. 2019; 231: 397–404. 10.1016/j.jenvman.2018.10.065 30368149

[38] Sanderson E, Forrest J, Loucks C, Ginsberg J, Dinerstein E, Seidensticker J, et al Setting Priorities for the Conservation and Recovery of Wild Tigers: 2005–2015. Washington, DC: Wildlife Conservation Society, World Wildlife Fund, Smithsonian, and Save the Tiger Fund; 2006.

[39] Laurance WF, Croes BM, Guissouegou M, Buij R, Dethier M, Alonso A. Impacts of roads, hunting, and habitat alteration on nocturnal mammals in African rainforests. Cons Biol. 2008; 22(3): 721–732. 10.1111/j.1523-1739.2008.00917.x 18477030

[40] Maynard RJ, Aall NC, Saenz D, Hamilton PS, Kwiatkowski MA. Road-edge effects on herpetofauna in a lowland Amazonian rainforest. Trop Conserv Sci. 2016; 9(1): 264–290.

[41] Mohd-Azlan J, Kaicheen SS, Yoong WC. Distribution, relative abundance and occupancy of selected mammals along paved road in Kubah National Park, Sarawak, Borneo. Nat Cons Res. 2018; 3(2): 36–46.

[42] Brodie JF, Giordano AJ, Ambu L. Differential responses of large mammals to logging and edge effects. Mamm Biol. 2015; 80: 7–13.

[43] Laurance WF, Yensen E. Predicting the impacts of edge effects in fragmented habitats. Biol Conserv. 1991; 55(1): 77–92.

[44] ESRI. ArcGIS Desktop: Release 10.5. Redlands, CA: Environmental Systems Research Institute 2017.

[45] Imron MA, Herzog S, Berger U. The influence of agroforestry and other land-use types on the persistence of a Sumatran tiger (*Panthera tigris sumatrae*) population: an individual-based model approach. Environ Manage. 2011; 48(2): 276–288. 10.1007/s00267-010-9577-0 20967444

[46] Shah P, Baylis K. Evaluating heterogeneous conservation effects of forest protection in Indonesia. PLOS ONE. 2015; 10(6): e0124872 10.1371/journal.pone.0124872 26039754 PMC4454437

[47] Jedrzejewski W, Niedzialkowska M, Nowak S, Jedrzejewska B. Habitat variables associated with wolf distribution and abundance in northern Poland. J Wildl Manage. 2002; 66: 1235–1245.

[48] Fuller TK. Population dynamics of wolves in north-central Minnesota. Wildl Monogr. 1989; 105: 1–41.

[49] Griffiths M, van Schaik CP. The impact of human traffic on the abundance and activity periods of Sumatran rainforest wildlife forest. Conserv Biol. 1993; 7(3): 623–626.

[50] Danielsen F, Beukema H, Burgess ND, Parish F, Bruhl CA, Donald PF, et al Biofuel plantations on forested lands: double jeopardy for biodiversity. Conserv Biol. 2009; 23(2): 348–358. 10.1111/j.1523-1739.2008.01096.x 19040648

[51] Yaap B, Magrach A, Clements GR, McClure CJW, Paoli GD, Laurance WF. Large mammal use of linear remnant forests in an industrial pulpwood plantation in Sumatra, Indonesia. Trop Conserv Sci. 2016; Oct-Dec: 1–13.

[52] Laurance WF, Clements GR, Sloan S, O’Connell CS, Mueller ND, Goosem M, et al A global strategy for road building. Nature. 2014; 513: 229–233. 10.1038/nature13717 25162528

[53] Hughes AC. Have Indo-Malaysian forests hit the end of the road? Biol Conserv. 2018; 223: 129–137.

